# Forensic Medicine

**Published:** 1872-10

**Authors:** Charles B. Knowlton

**Affiliations:** University of Buffalo. Medical Department


BUFFALO

Medical and Surgical Journal.

VOL XII. OCTOBER, 1872. No. 3

### Original Communications.

——:o:——

### ART. I.—*Forensic Medicine. Thesis.* By CHAS. B. KNOWLTON.

Published by vote of the Faculty.

Forensic Medicine, the science which applies the principles and practice of the different branches of medicine to the determination of doubtful questions in courts of justice. As the term implies, it is a mixed science, in which, the practitioners of both law and medicine have a common interest.

In speaking of the education of lawyers. Sir Edward Coke said, "Some knowledge of every science and art is not only useful but even necessary." Certainly, the medico-legal issues of the day render a practical knowledge of this branch indispensable to the legal profession.

This subject is of equal, if not of greater importance, to medical men, who in courts of law, act as representatives and exponents of medical knowledge and professional skill.

In the Medical Department of the University of Buffalo this science occupies a prominent place in the curriculum of instruction. Scarcely a lecture has been presented which did not embrace some items of interest in a medico-legal point of view, and in many instances they have been illustrated by reference to interesting and instructive trials, which involved facts inconclusive without the aid of medical testimony. Much has been said of the importance of basing such evidence upon medical learning and professional skill; and of the value of a practical knowledge of this science as essential to fit medical men for the duties imposed upon them by the exigencies of modern society.

In reviewing the history of Forensic Medicine, we find that the ancient Roman law-courts recognized the application of medical knowledge in the administration of laws. Their method consisted merely of referring doubtful medico-legal questions to the "authority of the learned Hippocrates." In tracing its progress we find that as the science of medicine progressed in development, this branch correspondingly attained the dignity of a science. The beginning of a system as now presented, dates from the commencement of the sixteenth century, since then, its importance has awakened much interest among medical men, and numerous works on this subject have been contributed, which do honor to the profession.

When presented as a system, the science of Forensic Medicine embraces various branches, which may be conveniently arranged under appropriate heads. In briefly discussing the subject, we will only consider the legal duties of medical men as experts and practitioners.

Medical men when called to testify on questions depending upon their professional knowledge, are termed medical experts. The term *experts* includes all persons of experience or professional skill in the science or practice in question.

As to what constitutes a *medical expert*, the law-courts do not seem to agree.

On the one hand it appears to be admitted that in questions of science or skill, the witness should not only be conversant with the subject matter, but also have experience or practice in the art or profession. While on the other hand it has been ruled that persons practicing as physicians, without medical diploma, or licence from an examining board, are to be considered as experts, on equal footing with the most thoroughly educated medical men.

It has been held that a witness who has studied a profession, although not engaged in practice, may testify as an expert. As long as the courts do not discriminate between a legitimate member of the profession, and one who merely assumes the title of Doctor, perhaps not even possessing the primary elements of a medical education, we can reasonably anticipate some dissatisfaction in regard to medical testimony.

It is evident that a medical expert, properly so called, is qualified to give opinions based upon practical knowledge and experience. Contrary to the general rule, that witnesses must confine themselves to facts, experts are allowed to state their opinions. As to the limit of opinions expressed by an expert in evidence, certain rules have been instituted, which confine such witnesses to state facts and opinions within the scope of their professions.

The opinions of medical experts may be asked upon supposed cases, similar to the one before the court, but the opinion of one expert is not admissible as to whether a certain state of facts was sufficient to justify another expert in the formation of an opinion which he had given in evidence.

The opinions of medical men are good evidence to go to a jury in actions to recover damages for personal injuries.

Where the defense is insanity, the medical expert may be called to give an opinion as to whether such and such appearances, in his judgment, are symptoms of insanity, and his opinions may be based upon facts proved by other witnesses, in this case, he must have heard all the evidence tending to prove insanity. It is doubted whether he could be asked his opinion as to whether the particular act with which the prisoner is charged is one of insanity, for this is a point to be decided by the jury.

It has been ruled that a medical witness is not competent to deliver an opinion on insanity unless he has had experience in the treatment of this disease. The wisdom of such a decision is apparent to all who appreciate the difficulties in the diagnosis of some forms of insanity. The statute provides that medical practitioners shall not be permitted to disclose any information confided to them by their patients in their professional capacity, and which information was necessary in order to prescribe as a physician, or to perform any act as a surgeon. It has been held that where physicians are consulted as means of procuring an abortion, they are not debarred, under the statute, from testifying, it being doubtful whether such a communication would be considered professional, within the meaning of the statute.

The nature of medical expert testimony is varied to a far greater extent than that of any other profession, science or art. Medical men are constantly called upon to testify as to the cause of disease, or of death, the consequence of wounds, and as to the sane or insane state of a person's mind. Within the sphere of medical expert testimony, questions arise which are vast and unlimited in their range, and many of them fathomless in their depth.

In order to meet the legal as well as the medical requirements in delivering an opinion before the court, the medical expert should comprehend the differences between medical and legal definitions, which owing to different objects, or intentions contemplated by medicine and law, respectively, are necessarily dissimilar. As an example, we will take the term *wounds*. In surgery the term means strictly. "A solution of continuity;" in law it means, "injuries of every description that affect either the hard or the soft parts, comprehending bruises, contusions, fractures, luxations etc." In surgery, wounds are classified as "incised, contused, lacerated and punctured." In law they are divided into "slight, dangerous and mortal." It is evident that a knowledge of these distinctions is of much importance to the witness in the examination; otherwise, advantage is afforded the cross-examiner.

The legal duties and responsibilities assumed by the physician or surgeon in the treatment of a patient, are "1. He shall exercise ordinary skill and diligence. 2. He shall devote ordinary care and attention." In the interpretation of the law, commentators explain the term "ordinary skill," as meaning ordinary *professional* skill. Professional skill meaning average skill as compared with the prevailing standard of skill of the profession; that which results from having the necessary degree of professional knowledge. In the requirement "to use ordinary care and diligence" the term "ordinary" has the same force as when used in reference to skill. The physician is to be governed by the gravity of the circumstances, the three qualities mentioned requiring him to use his best judgment in affecting a cure. Having exercised ordinary skill and care in the treatment of his patient, the medical practitioner is not considered responsible for want of success, nor for mistakes in cases of real doubt and uncertainty. Whenever it can be made to appear that the practitioner has failed to conform with the requirements, which we have briefly stated, he may be judged guilty of malpractice.

At this point it may be admissible to refer to the peculiar position of the practitioner as regards his liability to unjust accusations. Although in some cases conduct may deserve the penalty of the law, yet those who are familiar with the state of facts are aware that the greater proportion of actions for malpractice are unjustifiable.

It is evident that suits are often originated by unworthy motives, and it may be asserted that sometimes such cases are instigated by the promptings of those who claim alliance to the medical profession, at least, suits are often encouraged by opinions expressed by them, in regard to the professional services of another. Such indiscreet and inconsiderate opinions are by listeners often magnified in meaning and enlarged in substance until the practitioner is made the victim of a vexatious and perhaps, a ruinous suit.

In suits for malpractice, founded on the treatment of fractures, it is often found on sifting the facts that the bad result is wholly attributable to the carelessness of the patient when not under the control of his medical adviser. A popular error exists to quite an extent that whatever deformity results from a fracture is the fault of the surgeon. The differences in the nature and terminations of fractures are not understood. Many are not aware that oblique and compound fractures almost invariably unite at the expense of shortening or other disfigurement in spite of the most admirable surgery.

The following facts on this subject have been established after careful investigation, by the most prominent surgeons, "in fractures of the tibia and fibula, both compound and simple, perfect results are in the proportion of only one to about three of the cases treated; and, in fractures of the femur and clavicle, complete cure results in about one case in five; in fractures of the patella, a perfect cure happens only in one case in six." If the community at large would become educated to these facts, we are confident that the weight of public opinion would discountenance many of the ruinous and irritating conspiracies against medical practitioners.

In conclusion of this brief review of the duties of medical men, suffice it to say, that their obligations to courts of law; to medical science; and their professional bretheren are all of the greatest importance, and when ably performed, place the profession of medicine in an exalted rank in the eyes of the world.

University of Buffalo. Medical Department.
Dec. 30th, 1871.

